# Innovative use of Proximal Humeral Interlocking System (PHILOS) plate for fixation of osteoporotic fibula fracture

**DOI:** 10.1308/003588412X13373405386015g

**Published:** 2012-09

**Authors:** KS Wronka, C Topliss

**Affiliations:** Abertawe Bro Morgannwg University Health Board,UK

**Figure 1:**
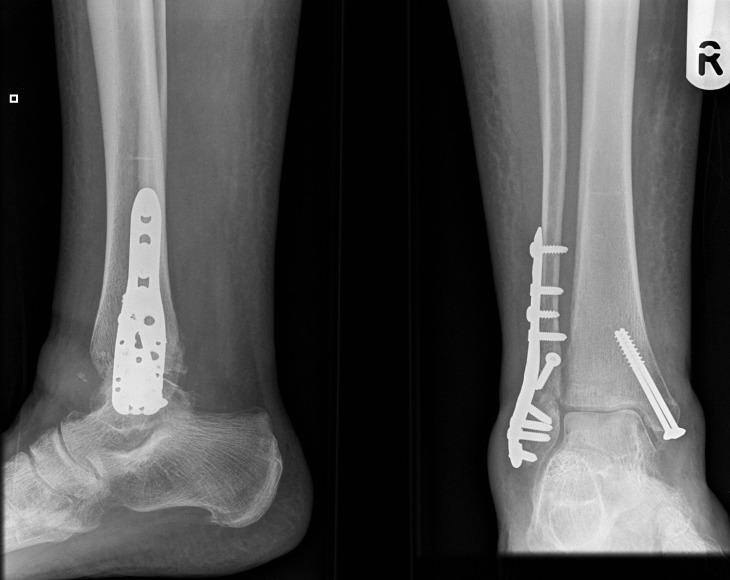
Plain x-rays showing ankle 12 weeks following fixation of distal fibula using PHILOS plate

Operative treatment of comminuted, osteoporotic distal fibula fractures is challenging due to poor bone stock. Conventional plates provide inadequate fixation. We describe treatment using the Proximal Humeral Interlocking System (PHILOS) plate (Synthes, Welwyn Garden City, UK). Following adequate exposure and reduction of the fracture, the PHILOS plate applied to the fibula in an inverted position. Proximal plate fixation using a compression screw is followed by distal fixation using locking screws. An image intensifier ensures satisfactory screw length. The PHILOS fixation is strong and provides angular stability. This technique has been used successfully in three patients with osteoporotic fibula fractures (Fig 1). The plate was not prominent and did not overlap with the distal fibula.

